# When ‘Clean’ is not clean: a diagnostic pitfall in Gram staining leading to pseudobacterial meningitis

**DOI:** 10.1099/acmi.0.001219.v3

**Published:** 2026-08-10

**Authors:** Takayuki Kawamura, Sachie Koyama, Haruka Karaushi, Yasuhiro Ebihara, Kotaro Mitsutake

**Affiliations:** 1 Department of Infectious Diseases and Infection Control, Saitama International Medical Center, Saitama Medical University, 1397-1 Yamane, Hidaka, Saitama, Japan; 2 Clinical Laboratory, Saitama Medical University International Medical Center, 1397-1 Yamane, Hidaka, Saitama, Japan; 3 Department of Laboratory Medicine, Saitama Medical University International Medical Center, 1397-1 Yamane, Hidaka, Saitama, Japan

**Keywords:** cerebrospinal fluid, diagnostic pitfall, Gram staining, pseudobacterial meningitis, standard operating procedure

## Abstract

**Introduction.** Gram staining is a fundamental and widely used technique in clinical microbiology because of its simplicity and diagnostic value, particularly in the diagnosis of bacterial meningitis. However, its reliability may be compromised by contamination and staining artefacts.

**Case Presentation.** This report describes a case of pseudobacterial meningitis in a previously healthy 25-year-old woman who presented with fever, headache and nuchal rigidity. Cerebrospinal fluid (CSF) analysis revealed pleocytosis with a predominance of mononuclear cells. Gram staining revealed findings consistent with Gram-negative bacilli, prompting empiric antibacterial therapy with concurrent antiviral treatment. Comprehensive microbiological investigations, including CSF culture and broad-range 16S rRNA PCR testing, yielded negative results. Residual material or microscopic debris on supposedly ‘clean’ slides was suspected to produce artefact-like appearances that mimicked micro-organisms, leading to false-positive interpretations.

**Conclusion.** This case highlights a potential laboratory pitfall in the interpretation of CSF Gram stains and underscores the importance of considering staining artefacts, particularly when Gram stain findings are discordant with the overall clinical and laboratory findings.

## Data Summary

This study is a single case report, and no original data were generated or required to support or reproduce the findings.

## Introduction

Gram staining is a fundamental and valuable technique in clinical microbiology, widely employed because of its simplicity and diagnostic efficacy, particularly in the diagnosis of bacterial meningitis using cerebrospinal fluid (CSF) [1, 2]. However, false-positive Gram stain results have been reported in 0.1%–1.3% of CSF specimens [3–5]. Reported causes of false-positive findings include contamination of instruments or reagents [6–11], stain precipitates [12, 13], slide-related artefacts (e.g. residual debris or scratches on glass slides) [14, 15] and environmental debris that can mimic bacterial morphology [5, 13]. Although glass slides are commercially supplied as ‘clean’, residual material or microscopic debris may remain on their surfaces and potentially contribute to false-positive findings. In this report, we present a case of pseudobacterial meningitis in which the CSF Gram stain revealed findings initially interpreted as Gram-negative bacilli but later suspected to represent artefact-like appearances rather than true micro-organisms. This case highlights a laboratory pitfall in the interpretation of CSF Gram stains and emphasizes the need to consider staining artefacts, particularly when Gram stain findings are discordant with the overall clinical and laboratory picture.

## Case presentation

A previously healthy 25-year-old woman presented to the emergency department with a progressively worsening headache and a 3-day history of fever. Physical examination revealed nuchal rigidity, and the patient was subsequently admitted to the hospital. On admission, her body temperature was 38.5 °C, and her Glasgow Coma Scale score was 15 (E4V5M6). Kernig’s sign was positive, and no other focal neurological abnormalities were observed.

### Laboratory and clinical information

**Fig. 1.**
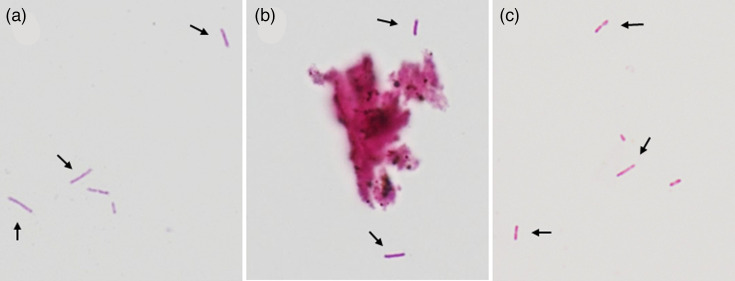
Comparative Gram stain findings in the patient specimen, an unused glass slide and a true Gram-negative bacillus control specimen. (**a**) CSF specimen obtained from the patient at the time of presentation. (**b**) Unused commercially supplied glass slide. (**c**) *Pseudomonas aeruginosa* used as a positive control representing a true Gram-negative bacillus. Arrows indicate representative Gram-negative bacillus-like appearances (original magnification ×1,000).

Laboratory findings on admission were as follows: a white blood cell count of 10,250 µl^−1^, with 81.8% neutrophils and 11.8% lymphocytes; a C-reactive protein level of 0.25 mg dl^−1^; and a procalcitonin level of 0.05 ng ml^−1^. Her blood glucose concentration was 118 mg dl^−1^. CSF analysis revealed pleocytosis (956 cells µl^−1^), with a predominance of mononuclear cells (91%) and 9% polymorphonuclear cells. CSF glucose and protein concentrations were 62 mg dl^−1^ and 108 mg dl^−1^, respectively. Gram staining was performed manually using a modified Bartholomew–Mittwer method and a commercially available staining kit (Muto Pure Chemicals Co., Ltd., Tokyo, Japan), which consisted of crystal violet staining, iodine treatment, acetone–ethanol decolourization and fuchsine counterstaining. The CSF Gram stain revealed findings that were initially interpreted as Gram-negative bacilli (Fig. 1). The slide was independently reviewed by two additional certified medical technologists with expertise in clinical microbiology. All three observers initially interpreted these findings as Gram-negative bacillus-like organisms. Ziehl–Neelsen staining and CSF PCR testing for tuberculosis yielded negative results.

Although the predominance of mononuclear leucocytes was not consistent with bacterial meningitis, meropenem therapy was initiated because the Gram stain suggested the presence of Gram-negative rods. Acyclovir was also administered as empiric antiviral therapy for suspected herpetic meningitis. By the second hospital day, the patient’s fever had resolved, and her neck stiffness had improved. CSF bacterial cultures were negative, and broad-range 16S rRNA gene PCR testing detected no bacterial or fungal pathogens. On hospital day 7, herpes simplex virus type 2 meningitis was diagnosed based on a positive CSF PCR result from the admission specimen. Subsequently, Gram staining of unused laboratory glass slides revealed microscopic findings resembling Gram-negative bacilli on all examined slides. These findings persisted even after ethanol immersion. Additional reagent and slide control experiments were performed to evaluate the Gram-staining reagents and glass slides as potential sources of the observed Gram-negative bacillus-like findings. Control staining procedures were performed using blank glass slides and Gram-staining reagents from different manufacturing lots. Gram-negative bacillus-like appearances were observed on blank slides from the implicated lot, even when Gram-staining reagents from different lots were used. However, these appearances disappeared after the slides were wiped with alcohol-soaked cotton before staining and were not observed on glass slides obtained from a different lot. Collectively, these observations suggested that the Gram-negative bacillus-like findings were more likely attributable to slide-surface contamination or debris than to contamination of the staining reagents.

In addition, the slide surfaces were swabbed for culture and subjected to broad-range 16S rRNA PCR testing; however, no micro-organisms were recovered by culture, and no significant amplification was detected by PCR. Although the exact cause could not be conclusively determined because the residual material was neither directly identified nor chemically analysed, the Gram-negative bacillus-like appearances observed on the CSF Gram stain were considered more likely to be attributable to residual material or microscopic debris on the glass slides.

Meropenem was subsequently discontinued, and the patient recovered without any complications. This case report was conducted in accordance with the ethical principles outlined in the Declaration of Helsinki. The case report was exempt from institutional review board (IRB) review under our hospital’s IRB regulations because it was a retrospective study conducted with full consideration for the protection of personal information.

## Discussion

Pseudobacterial meningitis is a rare phenomenon that has been reported in association with contamination, slide-related debris or staining artefacts [6–9]. Jacob and Stobberingh [15] reported that 22% of CSF specimens examined on slides that had not undergone alcohol-based cleaning showed contaminating micro-organisms, highlighting the potential impact of slide preparation on the interpretation of Gram stains.

Additionally, several reports have described the detection of micro-organisms in CSF specimens despite the absence of clinical evidence of meningitis. According to Olson and Hoeprich [16], of 182 CSF samples that tested positive for micro-organisms, 101 were obtained from patients without clinical evidence of meningitis, with *Staphylococcus epidermidis* and oral bacteria being among the organisms identified. Furthermore, false-positive microbiological findings associated with contaminated povidone-iodine antiseptics have also been reported [17]. Cunha and Cohen [14] described 11 cases in which micro-organisms were detected in CSF smears and cultures despite the absence of corresponding clinical manifestations. Contaminated tubes or transport media were suggested as the sources of contamination; however, only two cases involved issues related to glass slides.

Therefore, failure to wipe glass slides before use when performing CSF Gram staining may contribute to misleading or false-positive Gram stain interpretations. Although slide-surface debris remained a plausible explanation in the present case, other potential sources of artefact formation should also be considered. Stain precipitates may occasionally mimic bacterial morphology and thereby result in false-positive interpretations [5, 13]. Environmental contamination may also contribute to erroneous Gram stain interpretations by producing micro-organism-like appearances [6–9, 14]. In addition, contamination of staining reagents has been reported as a cause of pseudomeningitis and false-positive Gram stain findings in clinical specimens [11, 12]. Optical artefacts should also be considered a potential source of bacterial-like appearances observed during microscopic examination of Gram-stained specimens [18]. Because the exact origin of the observed findings could not be conclusively determined, none of these potential explanations can be completely excluded.

In laboratory settings, particularly during the handling of glass slides, it remains unclear whether microbiology technicians routinely wipe slides before use as part of standard laboratory practice. Although experienced laboratory technicians may assume that cleaning glass slides is standard practice, neither major Japanese laboratory manuals nor widely referenced international guidelines explicitly recommend that slides be wiped before use. The *ASM Manual of Clinical Microbiology* [19] states only that ‘clean’ slides should be provided, without specifically recommending a pre-use cleaning procedure.

In the present case, the technician who performed the Gram stain had recently been transferred to the microbiology laboratory, and the standard operating procedures (SOPs) specified only that slides should be ‘cleaned’, without clearly describing the appropriate cleaning method. Accordingly, the slides were cleaned by immersion in ethanol before use. Regardless of their exact origin, these observations underscore the importance of clearly defined and standardized slide preparation procedures.

Gram stain findings should be interpreted in conjunction with the available clinical information and CSF parameters and should be considered cautiously within the overall clinical context. Unexpected or discordant findings should prompt a careful review of the specimen and staining procedure, as well as timely communication with clinicians.

Proper slide preparation is essential for accurate Gram stain interpretation. The incorporation of a pre-use slide-wiping step into SOPs may help reduce the risk of slide-associated artefacts and false-positive findings. While many microbiological tests have transitioned to automated platforms [20, 21], awareness of potential artefacts and technical pitfalls remains important during microscopic interpretation. Even slides supplied as ‘clean’ may not always be free of residual materials capable of producing micro-organism-like appearances. In response to this case, the SOP for Gram staining at our institution has been revised accordingly to include a pre-use slide-cleaning procedure.

## Conclusion

Although the exact origin of the observed findings could not be conclusively determined, this case highlights a laboratory pitfall in the interpretation of CSF Gram stains. Awareness of potential staining artefacts, together with careful evaluation of unexpected or discordant Gram stain findings and adherence to clearly defined laboratory procedures, may help reduce the risk of misinterpretation.

## Abbreviations

CSF: cerebrospinal fluid
IRB: institutional review board
SOPs: standard operating procedures
